# Adult Exposures to Toxic Trace Elements as Measured in Nails along the Interoceanic Highway in the Peruvian Amazon

**DOI:** 10.3390/ijerph19106335

**Published:** 2022-05-23

**Authors:** Stacy M. Pettigrew, William K. Pan, James Harrington, Axel Berky, Elvis Rojas, Beth J. Feingold

**Affiliations:** 1 Albany College of Pharmacy and Health Sciences, Albany, NY 12208, USA; 2 Nicholas School of the Environment, Duke University, Durham, NC 27708, USA; william.pan@duke.edu (W.K.P.); axel.berky@duke.edu (A.B.); 3 RTI International, Research Triangle Park, NC 27709, USA; jharrington@rti.org; 4 Hospital Santa Rosa, Puerto Maldonado 17001, Peru; elvisrojasju@gmail.com; 5 Laboratorio Referencial de Madre de Dios, Puerto Maldonado 17001, Peru; 6 School of Public Health, University at Albany, Rensselaer, NY 12144, USA; bfeingold@albany.edu; 7 Institute for Health and the Environment, University at Albany, Albany, NY 12084, USA

**Keywords:** toxic trace elements, artisanal and small-scale gold mining, fish consumption, environmental exposures, Madre de Dios, Peru, Amazon rainforest

## Abstract

Deforestation, artisanal and small-scale gold mining (ASGM), and the rapid development related to highway expansion cause opportunities for toxic trace element exposure in the Amazon region of Madre de Dios (MDD), Peru, one of the most biologically diverse places in the world. The objective of this study was to assess the exposure to arsenic, cadmium, lead, and mercury among adults in Madre de Dios. In total, 418 adult (18+ years) participants in the Investigacion de Migracion, Ambiente, y Salud (IMAS) (Migration, Environment, and Health Study) participated in this study. Consent, survey data, and biospecimens were collected between August and November 2014. Nail elements were measured by inductively coupled plasma sector field mass spectrometry. Differences by selected individual and household characteristics and local land uses were tested using one-way ANOVAs and linear mixed models. Adults in ASGM-affected areas had higher nail arsenic and nail cadmium than their non-ASGM counterparts. Higher household fish consumption was positively associated with nail mercury and nail lead. The results indicate that adult exposure to arsenic, cadmium, lead, and mercury is heterogeneous across Madre de Dios, and the exposures related to ASGM communities and fish consumption suggest that exposures from artisanal and small-scale mining are environmentally widespread. Further investigation is warranted to ascertain potential health impacts.

## 1. Introduction

**Figure 1:**
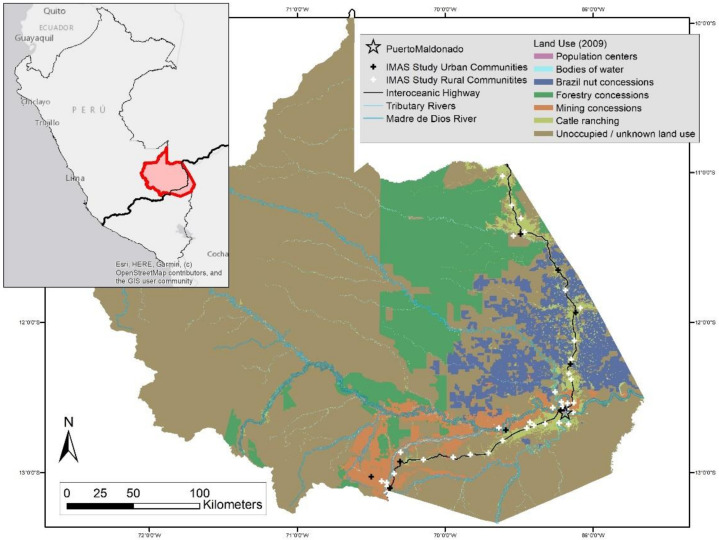
Land use in Madre de Dios, Peru; the Interoceanic Highway; and IMAS study sites (adapted from Feingold 2019).

Although lead (Pb), mercury (Hg), cadmium (Cd), and arsenic (As) are considered to be among the top ten chemicals of major worldwide public health concern, little monitoring of trace element contamination is ongoing in the Amazon rainforest, a sparsely populated and large area of dense, difficult terrain [1]. Madre de Dios (MDD), a region in the Peruvian Amazon, is experiencing rapid development, prompting growing concerns of environmental contamination and associated human health risks (Figure 1).

Prevalent in the western area of MDD, artisanal and small-scale gold mining (ASGM) is the largest source of anthropogenic Hg emissions in the world, accounting for an estimated 37% of global anthropogenic emissions [2]. ASGM exponentially increased in MDD as the price of gold rapidly rose after 2008, and Hg imports also increased [3]. The growth of mining in the region is also fueled by migration from the Andes and the paving of the Interoceanic Highway (IOH) [4]. Elevated Hg in MDD was measured in fish and suspended solids downstream from ASGM sites and in the hair of residents of mining-affected areas, as well as in ASGM communities around the world [3,4,5,6,7,8].

ASGM also accelerates the release of naturally occurring toxic elements into the soil as forests are disturbed [9,10]. Slash and burn techniques are widely practiced to clear the rainforest for agriculture and mining. Elevated levels of Hg, Cd, and Pb were measured in Amazonian soils after slash-and-burn agriculture in both Peru and Brazil [11,12]. Arsenic (As) released into the atmosphere by forest burning has also been identified as a source of dissolved As in the Amazon River [13]. From 1999 to 2012, ASGM was linked to the deforestation of an estimated 50,000 hectares of rainforest in MDD [14]. From 2012 to 2016, ASGM land use increased by 40%, penetrating into natural ecological reserves [15].

While Hg exposure from ASGM has garnered attention, co-exposures to other toxic trace elements (TTEs) may also be occurring. Hg and Pb are potent neurotoxins [16,17]. Pb also affects the cardiovascular and renal systems [18]. Cd and As are carcinogens [19,20], Cd is nephrotoxic, and As causes liver damage and neuropathy [20]. As and Cd in soil and Pb in water and plants were found and documented at ASGM sites in Brazil and Bolivia [21,22]. The Peruvian National Water Authority recorded concentrations of Hg, Pb, and/or As that exceeded national environmental standards (Estandares de Calidid Ambiental—ECA) in 4 out of 11 MDD locations where ASGM was prevalent [23]. Without data on other human exposures to environmental contaminates, authorities lack the information necessary to guide environmental health policies.

Non-invasive and easy to transport, nails are an advantageous biomarker medium in remote locations. Trace elements are deposited in the nailbed via the bloodstream [24]. Toenails have half the average growth rate of fingernails (1.62 mm/month for toenails and 3.47 mm/month for fingernails), taking 12–18 months to fully form, and thus reflect a longer exposure period [24,25,26]. Elevated concentrations of TTEs in nails in populations near environmentally contaminated areas were observed in numerous studies [27,28,29,30,31]. Elevated concentrations of TTE in nails were associated with negative health outcomes including increased blood pressure, hypertension, and mental stress [31,32].

To understand the nature and extent of exposure to TTEs along the IOH in the Peruvian Amazon, we leveraged data from the only population-based cohort in the region: *Investigacion de Migracion*, *Ambiente*, *y Salud (IMAS)* (Investigation of Migration, the Environment, and Health). The objective of IMAS was to conduct the first study to elucidate population level exposure to toxic trace elements in Madre de Dios using nails as a biomarker medium, and investigate associations with individual, household, and community-level variables. This analysis provides a baseline for environmental exposures in a biodiverse area undergoing rapid development.

## 2. Materials and Methods

IMAS was designed as a cross-sectional study with original data collection in 2011–2012. Follow-up data were collected in 2014. This study was approved by the Institutional Review Board (IRB) at the United States Naval Medical Research Unit, Detachment 6 (NAMRU-6) in Lima, Peru and the University at Albany—State University of New York.

The study population, chosen using two stage probability proportional to estimated size (PPES) sampling, spans the eight districts along the IOH in Madre de Dios; paving of the highway was completed in 2012 (Figure 1). The 2011–2012 two-stage study sample was stratified by urban/rural status. Urban communities were defined as those with 75 or more homes. The first stage selected 10 urban and 38 rural communities in Madre de Dios located within 10 km of the highway. The second stage selected households from these communities based on PPES, resulting in 18 households per urban community and 12 households per rural community. Two of the original forty-eight communities were mining communities that no longer existed at the start of data collection in 2011. Inclusion criteria required participants to have lived at their residence for at least 6 months prior to the date of the survey. Households with no adult available to consent after three visits were excluded. All household members (defined as those who regularly sleep in the home) were invited to participate in the study. Baseline data were collected from 1664 participants in 46 communities in 2011, with a response rate of 87% (78% completed surveys of 433 households).

In 2014, follow-up surveys were administered on both community and household levels, and data were collected from 310 households (1021 participants) in 46 communities, with a response rate of 64% (47% completed surveys from 309 households) (Supplementary Figure S1). Dropout was non-differential with respect to age, sex, and urban/rural status; however, households that did not participate in 2014 were more likely to be from Laberinto, one of several districts where small-scale mining is prevalent (Supplementary Table S1). Nail samples were collected from 418 adults. Those who did not give nail samples were more likely to be male, non-smokers, work either in the timber industry or drive a taxi, younger, and live in the districts of Huepetuhe and Inambari, where mining is common, than adult participants who gave nail samples (Supplementary Table S2). Paper surveys were administered by interviewers trained in research on human subjects. A community leader or longstanding community residents were interviewed for the community level survey, which included information on when the community was established, local population and migration, basic local infrastructure, land ownership, economic activities, and land use patterns, with attention to changes since administering the baseline survey in 2011. The economic head of household or their spouse was re-contacted for the 2014 household survey. The survey included questions on demographics, fertility, economics, household infrastructure, mobility, health, and household dietary information, as well as biospecimen (hair and nails) collection for dietary and elemental exposure assessment.

Statistical analyses were completed using SAS version 9.4 (2016, SAS Institute, Cary, NC, USA). Univariate summary statistics were computed for each trace element and other variables of interest at the individual, household, and community levels described below.

Individual demographics included age, sex, education level (some higher education/technical school, completed secondary, completed primary, less than primary/none/other), length of time in current district (>10 years, 5–10, <5 years), birth region (Andean, coastal, rainforest), smoking status, individual income from economic activities as well as any economic activity (agricultural, timber/logging, Brazil nut extraction, fishing, merchant/commercial, mining, professional/technical, employee of a store/restaurant/warehouse, taxi driver/transportation, other work).

Household characteristics included water source and disinfection, electricity source and cooking fuel used; mosquito controls; crops grown by the household (fruit, yuca, rice, cacao, meat/dairy); household income from economic activities; whether a member of the household worked as a miner, fisherman, or Brazil nut harvester; and frequency of consumption (never/monthly/weekly/daily) of various foods depending on the element, including fish, canned fish, meat, organ meats, yuca, yuca flour, fruit, and rice.

Community information included the district, community, and urban/rural status, as well as land use activities based on the most important sources of income for residents of the community and land usage in and adjacent to the community (agriculture, plantation agriculture, timber extraction, Brazil nut extraction, fishing, commercial activities, transportation services, livestock, gold mining/processing, hunting, and ecological reserves) as reported in the community level survey. In addition, whether community leaders reported the use of herbicides, pesticides, or chemical fertilizers was tested.

Nails were collected by study participants using stainless steel nail clippers and stored in an air-conditioned room in sealed envelopes until analyzed for total As, Cd, Hg, and Pb at the Trace Inorganic Laboratory, RTI International, Research Triangle Park, NC (USA). While toenails were preferred, fingernails were collected in place of toenails when necessary. Up to 10 nails per person were combined into a bulked sample to attain the minimum mass required for analysis. The sample was cleaned to remove external organic and inorganic contaminants using a method based on the IAEA nail cleaning method, first using HPLC grade acetone (Honeywell Scientific, Charlotte, NC, USA) in an ultrasonic bath, followed by a 0.1% solution of Triton X-100 surfactant (98% for molecular biology, Acros Organics, Geel, Belgium) in an ultrasonic bath (Crest Ultrasonic), and then rinsed with ~18 MΩ quality deionized water from a Milli-Q Element (Burlington, MA, USA) [33]. Samples were prepared by two-stage acid digestion using a graphite heating block (SCP Science, Quebec, Canada) at 95 degrees Celsius. To stabilize Hg, samples were spiked with a commercially available 1000 ug/mL stock standard of gold (High Purity Standards, Charleston, SC, USA) to produce a final concentration of 2 mg/L gold [34]. The first digestion stage used nitric and hydrochloric acid (Ultrex-purity grade; J.T. Baker Scientific, Radnor, PA, USA), and 30% hydrogen peroxide (Suprapur purity non-tin-stabilized, J.T. Baker Scientific) was added during the second stage. The samples were then diluted and analyzed for total Pb, Hg, Cd, and As using a high-resolution Inductively Coupled Plasma Sector Field Mass Spectrometer (Element 2, Thermo Science, Bremen, Germany) to obtain the greatest sensitivity for all elements, while eliminating analytical interferences. Aqueous calibration solutions were prepared using National Institute of Standards and Technology (NIST) traceable 10 µg/mL single-element stock solutions (High Purity Standards) for each analyte. Limits of detection (LOD) for each element were established by multiplying the standard deviation of the quality control blank measurements by the appropriate Student’s t-value at a 99% confidence interval [35]. Due to low sample masses, method blank (acid digestions containing no nails) and spiked-method blank samples (acid digestions without nails and spiked with three different known concentrations of each element) were used as quality control samples to assess the contribution of background signals to measured elements and to assess the accuracy of the elements passed through the sample digestion process. Relative errors for the elements across all three concentration levels were 3% for As, 3% for Cd, 0.42% for Hg, and 0.7% for Pb, demonstrating minimal influence of the digestion process on the elemental recovery. Two samples per batch were re-analyzed for precision.

Correlations between different elemental concentrations in nails were estimated using the Pearson correlation coefficient. Outliers were identified as concentrations ± 1.5 times the 25–75th interquartile range but were not removed. Extreme outliers were defined as 4 times the standard deviation above the mean and were reassigned ranked values beginning at two times the standard deviation above the mean with proportionate percentage increases. Three individuals’ nail Pb concentrations were extreme outliers. The natural logarithm of each element was taken to produce normally distributed data. Log-element concentrations were used in bivariate and multivariate analyses. Differences in log trace element means were individually tested by one-way ANOVAs for each variable described above. For each element, all characteristics with a significant one-way ANOVA association at a *p*-value ≤ 0.05 were tested for correlations by Spearman rank coefficients. For pairs with coefficients greater than 0.60, the variable with the smaller ANOVA *p*-value was kept in the model. The multivariate influence of all significant characteristics on concentrations of each trace element was tested using mixed linear models with a variance component covariance structure and restricted maximum likelihood (REML) estimation.

## 3. Results

Only 39% of participants were born in the Amazon rainforest region, 42% in the Andes, 6% in coastal regions, with the remainder missing birthplace data (Table 1). Additionally, 22% of participants moved to their current district within the past ten years. Agricultural work was the primary activity of those that reported earning an income. While 24% of individuals lived in communities proximal to ASGM activities, only 9 persons (2%) reported working in gold mining. Municipal water was the most common source of household drinking water, followed by well water and surface water (Table 2). In terms of occupations, 15 individuals lived in a household with a member who reported a fishing occupation, 25 with a member who reported working in Brazil nut harvesting, and 22 with a miner. Livestock and agriculture were the most commonly reported adjacent land uses throughout the region (Table 3).

While some r-values were statistically significant, Pearson correlation coefficients between individual trace elements were all below 0.30 (Table 4). One participant was missing household data and was not included in multivariate analyses.

### 3.1. Arsenic

Total nail As ranged from 8 to 1260 ng/g, with a median of 177 ng/g. Nineteen subjects were defined as upper outliers, with nail As ranging from 445 to 1260 ng/g.

In the multivariate analysis, females had significantly lower As concentrations than males, as did persons completing secondary school compared to those only completing primary school, while individuals born in coastal areas tended to have higher total nail As compared to the rainforest (Table 5). Persons living in communities proximal to mining activities were also associated with increased As, while proximal timber harvesting and cattle raising were associated with decreased As. Proximal timber harvesting was highly correlated (r = 0.66, *p* < 0.0001) with proximal Brazil nut harvesting; only the former was included in the model. Similarly, a household member working in a fishing occupation was highly correlated with an individual fishing occupation (r = 0.81, *p* < 0.0001).

### 3.2. Cadmium

Total nail Cd ranged from 1 to 3710 ng/g, with a median of 62 ng/g. Thirty-three subjects were identified as upper outliers with nail Cd over 222 ng/g. In a multivariate mixed linear regression, persons born in the Andes or coastal regions had significantly higher nail Cd than those born in the rainforest, while persons in households that reported eating fish or yuca daily had lower nail Cd compared to those who ate it monthly and weekly, respectively (Table 6). People living in communities proximal to ASGM also had significantly higher Cd. Fishing occupation was highly correlated with household member works in fishing occupation (r = 0.81, *p* < 0.0001). Only the former was included in the model.

### 3.3. Mercury

Total nail Hg ranged from 18 ng/g to 5600 ng/g, with a median of 512 ng/g. There were 27 upper outliers, with total Hg concentrations from 1870 ng/g to 5600 ng/g. In the multivariate analysis, any amount of household fish consumption compared to none was strongly associated with increased nail Hg (Table 7). Fishing occupation and living in a household where a member of the household worked in a fishing occupation were highly correlated (0.81, *p* < 0.0001). Only the household variable was included in the model and was significantly associated with increased Hg (β = 0.71, *p* = 0.0436). Similarly, individual mining occupation and living in a household where a member of the household worked in a mining occupation were also highly correlated (0.63, *p* < 0.0001), and only the household level variable was included in the multivariate model. It was also positively associated with increased Hg, although it failed to reach statistical significance at a *p* < 0.05 level (β = 0.42, *p* = (*p* = *0*.0790)). In addition, although proximal timber harvesting was highly correlated (r = 0.66, *p* < 0.0001) with proximal Brazil nut harvesting, only the former was included in the model.

### 3.4. Lead

Total nail Pb concentrations ranged from 112 ng/g to 572,000 ng/g, with a median of 734 ng/g. There were 53 upper outliers (13% of participants), with Pb concentrations ranging from 2870 ng/g to 527,000 ng/g. Three individuals’ nail Pb concentrations were reassigned ranked values (120,000 to 59,791; 158,000 to 70,355; 527,000 to 74,082). In the multivariate regression, female sex was inversely associated with log Pb (Table 8). Coastal and Andean birthplaces were positively associated with log Pb concentrations compared to the rainforest. As fishing occupation and living in a household where a member of the household worked in a fishing occupation were highly correlated (r = 0.81, *p* < 0.0001), only the household variable was included in the model. It was significantly associated with increased nail Pb. Daily household fish consumption was also associated with increased lead compared to no consumption. Living in a household where a member reported working in Brazil nut harvesting was inversely associated with nail Pb (*p* = 0.0510).

## 4. Discussion

Concentrations in all four TTEs indicate environmental routes of exposure. Unlike numerous other analyses, gold mining occupation and living in a household with a miner were not significantly associated with elevated total nail Hg analysis in the multivariate analysis [7,36]. The significant positive association with living with a fisherman and the strong positive associations with increased household fish consumption lend further evidence to widespread environmental exposure sources, rather than occupational ASGM exposures. This finding is consistent with studies documenting elevated Hg in fish downstream from gold mining activities and hair analysis of the same population, as Hg used in ASGM is converted to methylmercury in river sediment and biomagnifies up the aquatic food chain [24,36]. Increased levels of total nail Pb were also associated with daily household fish consumption, and living in a household where a member works in a fishing occupation was significantly associated with increased Pb and As. While the artisanal production and use of Pb sinkers present a potential route of exposure from fishing, the Peruvian National Water Authority also reported elevated As, Hg, and Pb in water samples from rivers where mining is prevalent in MDD [23,37,38]. While IMAS did not gather data on wild game, a previous study in the region found lead and mercury exposures to be highly correlated, potentially due to the codependence on wild game shot with lead ammunition and wild fish [39]. Additionally, proximal mining activities were associated with increased total nail As and Cd, but were not significantly higher in individuals who reported mining as an occupation, consistent with environmental exposures and other regional studies documenting co-exposures to TTEs around mining activities [21,22]. It is also possible that TTE exposure profiles of different tasks within the mining occupation are not homogeneous.

Compared to persons born in the rainforest region, persons born in coastal regions had higher As, and those born in both coastal and Andean regions had higher Cd and Pb. These associations are not artifacts of fishing or fish consumption, as the vast majority of persons who lived in a household with a fisherman were born in the rainforest, as were people in households with the most frequent fish consumption (Supplementary Table S3). However, persons born on the coast were more likely to use well water daily, which was associated with increased nail As. While individuals born in the Andes were more likely to live near mining activities, the distribution of persons working in the mines was similar by birth region. It is also possible that the observed differences in exposures are due to unmeasured cultural differences.

Tobacco is a common source of non-occupational Cd exposure [17]. In this analysis, current smokers were more likely to be in the lower exposure quartile (data not shown). It is possible that this lack of association is due to relatively low observed Cd levels. While associations between nail Cd and smoking were observed in an Egyptian study (nail Cd mean 1.35 µg/g), the median from the current analysis (0.062 µg/g) is closer to other analyses in the US (0.02 µg/g) and Pakistan (0.05 ug/g), which did not observe these associations [40,41,42]. The discrepancy may also indicate that nails are not an accurate biomarker medium of cadmium exposures, particularly when compared to urine [43].

A major strength of this analysis is that it is the first report of integrated exposure to multiple TTEs in Madre de Dios. The IMAS Study is one of the first analyses to capture data from this remote location experiencing rapid change. The use of nails as a biomarker medium is beneficial for remote data collection; however, the lack of international clinical reference values and established population ranges limit comparisons. While population-based biomonitoring established baseline levels of comparison for metals and other exogenous contaminants in blood and urine, concentration in nails was not given equal examination. External contamination could also influence results despite cleaning techniques that were developed to differentiate exogenous concentrations [27,44]. While IMAS preferentially collected toenails to minimize potential external contamination, participants were more willing to give fingernail samples (Supplementary Table S4). In total, 96% of the 27 Hg outlier samples contained fingernails, and the 3 concentrations of Pb that were adjusted in the analysis were fingernail samples. As the type of nail did not significantly differ by Pb outlier status, it is possible that contamination was largely limited to these samples. Measured As concentrations were significantly higher in toenails than fingernails, possibly due to additional elemental deposition during the longer growth period. It is possible that combining finger and toenails into the same analysis limited the accuracy of results.

Repeat analysis of several split samples (Supplementary Table S5) demonstrated in some cases high variability, particularly for Cd and Pb. However, this may be attributed to variations in trace element deposition between different toes or fingers. Heterogeneity of Pb, Hg, and Cd in nails are consistent with the findings of a recent systematic review on their use as a biomarker medium [45]. These are good baseline findings due to the ease of collecting nails and high participant acceptance, but the literature indicates that other biomarker mediums such as blood and urine may be preferable for the monitoring of specific elements, which could benefit future studies on this topic.

The use of household level data to draw inferences about individual trace element concentrations is also a limitation. For example, household level information on diet could have missed individual differences and extremes between two different household members, causing non-differential misclassification and driving the results toward being null.

Although the original baseline study sample was determined using two stage probability proportional to estimated size (PPES) sampling, sampling weights were not used in this analysis as the representation calculated by PPES could not be guaranteed due to loss to follow-up. Participation was differential in respect to areas where small-scale gold mining is prevalent, perhaps due to high rates of short- and long-term migration in these areas, the largely illegal nature of such activities, potential distrust of outsiders, or a disbelief that TTEs can harm health. Despite this, we observed that exposures to As, Cd, and Hg were all strongly associated with nearby and adjacent mining activities, and thus warrant further investigation and protective public health action.

As the first study in the region examining TTEs in nails, our work provides insight into environmental health exposure risks in Madre de Dios. Concentrations of Cd and Pb were higher in this study population than in an indigenous Canadian population living near a closed gold mine, and the median Hg in this population was over three times the median Hg of a Nicaraguan gold mining community [31,32]. While the results cannot be used in clinical diagnoses, the range of concentrations and covariate data collected by the survey provide an opportunity to test potential exposure routes. This offers a feasible approach to intra-population comparisons and risk identification.

## 5. Conclusions

In this investigation into TTE concentrations measured in the nails of individuals living along the IOH in Madre de Dios, Peru, living in areas in and adjacent to mining activities were associated with exposure to As and Cd. Fishing was associated with higher As, Hg, and Pb concentrations, while more frequent household fish consumption was associated with higher concentrations of Hg.

While no clinical reference values for TTEs in nails exist to infer health implications or clinical diagnoses, these associations raise concern that environmental exposures to health-relevant toxic elements exist in the Peruvian Amazon and warrant further examination. Future analyses should include an assessment of children’s exposures, collect biomarkers in gold-standard mediums such as blood and urine to facilitate comparisons with other work, and couple human exposure and health assessments with environmental assessments to elucidate any potential environmental related sources of exposure to toxic elements.

## Figures and Tables

**Table 1:** Selected individual characteristics of persons who provided nail samples from the study population of IMAS (2014), Madre de Dios, Peru.

Characteristic	n	%
Total	418	100%
Age		
Missing	5	1%
Under 30	69	17%
30–44	123	29%
45–59	126	30%
Over 60	95	23%
Sex		
Female	218	52%
Male	200	48%
Smoking Status		
Non-Smoker	294	70%
Former Smoker	42	10%
Current Smoker	80	19%
Missing	2	0%
Length of Time in Current District		
>10 years	325	78%
6–10 years	51	12%
≤5 years	42	10%
Birth Region		
Missing	53	13%
Rainforest	164	39%
Andean	176	42%
Coastal	25	6%
BMI		
Missing	17	4%
Underweight	6	1%
Normal	131	31%
Overweight	159	38%
Obese	105	25%
Reported Primary Economic Activities		
Agricultural	127	30%
Merchant/Commercial	25	6%
Store/Restaurant/Warehouse Employee	20	5%
Brazil Nut Extraction	18	4%
Logging	18	4%
Taxi/Transportation	14	3%
Professional/Technical	10	2%
Fishing	9	2%
Gold Mining	9	2%
Other Job	41	10%
Reported Self-Earned Annual Income (Peruvian New Soles—Quartiles)		
None	158	38%
200–3000	64	15%
3500–7200	69	17%
7680–12,000	73	17%
12,480–67,500	54	13%
Education Level		
Less than Primary/None/Other	112	27%
Completed Primary	162	39%
Completed Secondary	107	26%
Higher Education/Technical	37	9%

**Table 2:** Selected household characteristics of persons who provided nail samples from the study population of IMAS (2014), Madre de Dios, Peru.

Characteristic	n	%
Household Water Source		
Municipal Water	159	38%
Public Pipe	9	2%
Protected Well	83	20%
Unprotected well	34	8%
Surface Water	103	25%
Rainwater	6	1%
Bottled Water	6	1%
Water Truck	16	4%
Other	1	0%
Disinfect Water		
Yes	270	65%
Sometimes	46	11%
No	101	24%
Disinfection Method Used		
Boil	197	62%
Chlorine	116	37%
Let Sit	3	1%
Electric Source		
None	85	20%
Grid	251	60%
Generator	81	19%
Primary Cooking Fuel		
Propane	192	46%
Wood	145	35%
Charcoal	45	11%
Electricity	15	4%
Agricultural Residues	12	3%
Kerosene	3	1%
Do Not Cook	5	1%
Reported Mosquito Control Strategies		
Treated Nets	182	44%
Use Insecticide	117	28%
Burn Garbage for the Smoke	78	19%
Burn Oil	22	5%
Sprayed Curtains	7	2%
Use Insect Soap	4	1%
Household Crops		
Fruit	109	26%
Yuca	66	16%
Rice	42	10%
Meat/Dairy	13	3%
Cacao	3	1%
Household Income from Economic Activities (Peruvian New Soles—Quartiles)		
No Income	13	3%
200–5100	99	24%
5200–9600	108	26%
9700–16,800	97	23%
16,900–72,000	101	24%
Member of the Household Works as		
Fisherman	15	4%
Brazil Nut Harvester	25	6%
Miner	22	5%
Household Fish Consumption		
Never	16	4%
Monthly	182	44%
Weekly	171	41%
Daily	48	12%
Household Canned Sardines Consumption		
Never	254	61%
Monthly	19	5%
Weekly	82	20%
Daily	62	15%
Household Canned Tuna Consumption		
Never	102	24%
Monthly	49	12%
Weekly	173	41%
Daily	93	22%
Household Fruit Consumption		
Never	8	2%
Monthly	42	10%
Weekly	187	45%
Daily	180	43%
Household Yuca Consumption		
Never	22	5%
Monthly	90	22%
Weekly	156	37%
Daily	149	36%

**Table 3:** Selected community characteristics of persons who provided nail samples from the study population of IMAS (2014), Madre de Dios, Peru.

Characteristic	n	%
Location		
Rural	288	69%
Urban	130	31%
District		
Huepetuhe	15	4%
Inambari	54	13%
Laberinto	33	8%
Tambopata	101	24%
Las Piedras	118	28%
Tahuamanu	37	9%
Iberia	23	6%
Inapari	37	9%
Proximal Activities		
Brazil Nut Harvesting	145	35%
Plantation Agriculture	225	54%
Agriculture	303	72%
Timber Harvesting	229	55%
Mining	101	24%
Commercial Activities	62	15%
Transportation Services	55	13%
Fishing	53	13%
Cattle Raising	361	86%
Ecological Reserves	87	21%
Use of Herbicides	177	42%
Use of Pesticides	166	40%
Use of Chemical Fertilizers	105	25%

**Table 4:** Pearson correlation coefficients of logged trace element concentrations in nails from the study population of IMAS (2014), Madre de Dios, Peru (n = 418).

	Arsenic r (*p*-Value)	Cadmium r (*p*-Value)	Mercury r (*p*-Value)	Lead r (*p*-Value)
Arsenic	1	0.20 (<0.0001)	0.07 (0.1659)	0.22 (<0.0001)
Cadmium		1	−0.02 (0.6316)	0.30 (<0.0001)
Mercury			1	0.06 (0.1895)
Lead				1

**Table 5:** Log total nail arsenic and characteristics with ANOVA *p*-values ≤ 0.05 of persons who provided nail samples from the study population of IMAS (2014), Madre de Dios, Peru (n = 417).

Parameter		ANOVA *p*-Value	Estimate	Standard Error	Pr > |t|
Intercept			5.7478	0.1717	<0.0001
Individual Characteristics					
Female Sex (v. male)	Female	<0.0001	−0.2848	0.0620	<.0001
Birth Region	Missing	0.0033	−0.0557	0.1009	0.5817
	Andean		0.1142	0.0753	0.1317
	Coastal		0.4882	0.1377	0.0005
	Rainforest		ref		
Education	Less than Primary/None/Other	0.0114	0.0503	0.0819	0.5402
	Completed Secondary		−0.1559	0.0812	0.0568
	Higher Education/Technical		0.1016	0.1275	0.4269
	Completed Primary		ref		
Fishing Occupation (v. not)		0.0280			
Professional Occupation (v. not)		0.0307	−0.4016	0.2187	0.0685
Household Characteristics					
Electricity	Grid	0.0148	−0.1603	0.1034	0.1235
	Generator		0.0197	0.1106	0.8593
	None		ref		
Use Well Water for Drinking (v. not)		0.0476	−0.1826	0.0899	0.0441
Use Surface Water for Drinking (v. not)		0.0186	−0.0546	0.1011	0.5904
How Water Is Disinfected	Boil	0.0443	−0.0491	0.0830	0.5555
	Chlorine		0.0878	0.0900	0.3310
	Do not Disinfect/Let Sit		ref		
Burn Garbage for Smoke for Mosquito Control (vs. not)		0.0094	−0.1043	0.0859	0.2266
Household Fruit Crop (v. not)		0.0016	0.0494	0.0869	0.5710
Household Yuca Crop (v. not)		0.0038	0.1326	0.1050	0.2086
Household Livestock (v. not)		0.0041	−0.3684	0.2069	0.0773
Household Member Works in Fishing Occupation (v. none)		<0.0001	0.3482	0.1917	0.0716
Community Characteristics: Proximal Activities					
Brazil Nut Harvesting (v. not)		<0.0001			
Agriculture (v. not)		0.0009	−0.1712	0.0888	0.0560
Timber Harvesting (v. not)		<0.0001	−0.1748	0.0768	0.0243
Mining (v. not)		<0.0001	0.2834	0.0867	0.0014
Cattle Raising (v. not)		0.0004	−0.2591	0.1014	0.0118

**Table 6:** Log total nail cadmium and characteristics with ANOVA *p*-values ≤ 0.05 of persons who provided nail samples from the study population of IMAS (2014), Madre de Dios, Peru (n = 417).

Parameter		ANOVA *p*-Value	Estimate	Standard Error	Pr > |t|
Intercept			4.2562	0.2275	<0.0001
Individual Characteristics					
Birth Region	Missing	<0.0001	0.09326	0.1425	0.5138
	Andean		0.4242	0.1059	<.0001
	Coastal		0.5492	0.1926	0.005
	Rainforest		ref		
Fishing Occupation (v. not)		0.0239			
Household Characteristics					
Household Member in Fishing Occupation (v. none)		0.0039	0.2218	0.3107	0.4765
Electricity Source	Grid	0.0372	−0.1931	0.1434	0.1804
	Generator		−0.07669	0.1543	0.62
	None		ref		
Uses Surface Water for Drinking (v. other source)	Yes	0.0341	−0.1516	0.1256	0.2294
Uses Insecticide for Mosquito Control (v. not)	Yes	0.0409	0.0277	0.1119	0.8049
Household Consumption Frequency of Fish	Never	0.0028	−0.3407	0.2595	0.1913
	Monthly		ref		
	Weekly		−0.04136	0.1066	0.6987
	Daily		−0.4056	0.1822	0.0276
Household Consumption Frequency of Canned Tuna/Fish	Never	0.0005	−0.1502	0.126	0.2352
	Monthly		−0.2814	0.1716	0.1032
	Weekly		ref		
	Daily		−0.08846	0.1284	0.4922
Household Consumption Frequency of Yuca	Never	0.0009	−0.3717	0.2302	0.1085
	Monthly		−0.08058	0.1304	0.5377
	Weekly		ref		
	Daily		−0.319	0.1174	0.0074
Community Characteristics					
In or Adjacent Agriculture (v. none)		0.0009	−0.04365	0.1365	0.7496
In or Adjacent Timber Harvesting (v. none)		0.0004	0.1229	0.119	0.3036
In or Adjacent Mining (v. none)		0.313	0.1262	0.0143	0.0181
In or Adjacent Commercial Activities (v. none)		0.0018	0.1997	0.1499	0.185
Rural (v. urban)		0.0357	0.09931	0.1255	0.4302

**Table 7:** Log total nail mercury and characteristics with ANOVA *p*-values ≤ 0.05 of persons who provided nail samples from the study population of IMAS (2014), Madre de Dios, Peru (n = 417).

Parameter		ANOVA *p*-Value	Estimate	Standard Error	Pr > |t|
Intercept			5.3975	0.4638	<0.0001
Individual Characteristics					
Age (years)		0.0008	−0.0037	0.0025	0.1462
Birth Region	Missing	0.0004	0.1685	0.1298	0.1963
	Andean		−0.1585	0.0985	0.1099
	Coastal		−0.0430	0.1695	0.8003
	Rainforest		ref		
Education	Less than Primary/None/Other	0.0052	−0.0517	0.1051	0.6236
	Completed Secondary		0.0970	0.0984	0.3259
	Higher Education/Technical		−0.0218	0.1497	0.8843
	Completed Primary		ref		
Brazil Nut Harvesting Occupation (v. not)		0.0397			
Fishing Occupation (v. not)		0.0101			
Agricultural Occupation (v. not)		0.0383	−0.0408	0.0842	0.6289
Mining Occupation (v. not)		0.0352			
Household Characteristics					
Cooking Fuel	Do Not Cook	0.0370	0.1413	0.4819	0.7698
	Electricity		0.4175	0.2747	0.1309
	Kerosene		−0.7900	0.5951	0.1865
	Charcoal		−0.0902	0.1765	0.6100
	Wood		0.0607	0.1255	0.6292
	Agricultural Residues		−0.0803	0.3475	0.8176
	Propane		ref		
Raise Livestock (v. not)		0.0072	0.2869	0.3191	0.3701
Household Member Works in Brazil Nut Harvesting Occupation (v. none)		0.0332	0.3338	0.2133	0.1199
Household Member Works in Mining Occupation (v. none)		0.0063	0.4230	0.2390	0.0790
Household Member Works in Fishing Occupation (v. none)		0.0008	0.7091	0.3481	0.0436
Household Consumption Frequency of Fish	Never	<.0001	ref		
	Monthly		1.0729	0.2640	<0.0001
	Weekly		1.1642	0.2627	<0.0001
	Daily		1.1254	0.3072	0.0004
Household Consumption Frequency of Fruit	Never	0.0044	ref		
	Monthly		−0.3464	0.3980	0.3856
	Weekly		−0.0852	0.3741	0.8203
	Daily		0.0696	0.3778	0.8540
Community Characteristics: In or Adjacent Activities					
Brazil Nut Harvesting (v. not)		0.0316	−0.1391	0.1161	0.2328
Timber Harvesting (v. not)		0.0415			
Commercial Activities (v. not)		0.0458	0.2093	0.1488	0.1618
Ecological Reserves (v. not)		0.0014	−0.2374	0.1274	0.0646

**Table 8:** Log total nail lead and characteristics with ANOVA *p*-values ≤ 0.05 of persons who provided nail samples from the study population of IMAS (2014), Madre de Dios, Peru (n = 417).

Parameter		ANOVA *p*-Value	Estimate	Standard Error	Pr > |t|
Intercept			6.5415	0.304	<0.0001
Individual Characteristics					
Female Sex (v. male)		0.0014	−0.3289	0.1013	0.0015
Birth Region	Missing	0.014	−0.02046	0.1726	0.9058
	Andean		0.243	0.1245	0.053
	Coastal		0.6948	0.2318	0.0032
	Rainforest		ref		
Fishing Occupation	Yes	0.0023			
	No				
Household Characteristics					
Member of Household Works in Fishing Occupation (v. none)	Yes	0.0014	0.7416	0.3515	0.0366
Member of Household Works in Brazil Nut Extraction (v. none)	Yes	0.023	−0.4696	0.2386	0.051
Household Consumption Frequency of Fish	Never	0.045	ref		
	Monthly		0.3097	0.2929	0.2921
	Weekly		0.2538	0.2964	0.3933
	Daily		0.6844	0.3435	0.0482

## Data Availability

Not applicable.

## Supplementary Materials

The following supporting information can be downloaded at: https://www.mdpi.com/article/10.3390/ijerph19106335/s1, Table S1: Adult participants in IMAS at baseline (2011) compared to follow up (2014), Madre de Dios, Peru; Table S2: Characteristics of Adults in the IMAS Study Population (2014) who provided nail samples compared to those who did not, Madre de Dios, Peru; Table S3: Individual Birth Region by Selected Characteristics; Table S4: Trace Element Concentrations and Differences in Trace Element by Nail Type for Adults in the IMAS Study Population, Madre de Dios, Peru (n = 399); Table S5: Trace Element in Nails Retest Percent Differences, Adults in the IMAS Study Population, Madre de Dios, Peru; Figure S1: Study Flow Chart.
