# Supplementary material for: Adult Exposures to Toxic Trace Elements as Measured in Nails along the Interoceanic Highway in the Peruvian Amazon
Source: Int J Environ Res Public Health. 2022 May 23;19(10):6335. doi: 10.3390/ijerph19106335 (PMC9142029; doi:10.3390/ijerph19106335)
Supplement: Supplementary file 1 [file ijerph-19-06335-s001.zip › ijerph-1697078-supplementary.pdf]

Adult Exposures to Toxic Trace Elements as Measured in Nails along the Interoceanic Highway in the Peruvian Amazon

Supplementary Tables

Table S1. Adult participants in IMAS at baseline (2011) compared to follow up (2014), Madre de Dios, Peru.

Characteristic	2011 Sample	2014 Sample		Dropped Out		Chi Sq. P-Value
	n	n	%	n	%	
Female	853	493	58%	360	42%	0.1559
Male	969	528	54%	441	46%	
Age (years)				(T-Test P-Value)		0.1435
Rural Community	1191	657	55%	534	45%	0.3020
Urban Community	631	364	58%	267	42%	
District						
Huepetuhe	118	70	59%	48	41%	< .0001
Inambari	284	160	56%	124	44%	
Laberinto	255	91	36%	164	64%	
Tambopata	354	220	62%	134	38%	
Las Piedras	474	274	58%	200	42%	
Tahuamanu	164	97	59%	67	41%	
Iberia	73	45	62%	28	38%	
Inapari	100	64	64%	36	36%	

Table S2. Characteristics of Adults in the IMAS Study Population (2014) who provided nail samples compared to those who did not, Madre de Dios, Peru.

Characteristic	Total	Gave nails		Didn't give nails		Chi Sq. P-Value
	n	n	%	n	%	
Female	309	218	71%	91	29%	0.0016
Male	341	200	59%	141	41%	
Smoking Status						
Non-Smoker	482	294	61%	188	39%	0.0083

Former Smoker		57	42	74%	15	26%	
Current Smoker		107	80	75%	27	25%	
**Age**							
Under 30		119	69	58%	50	42%	0.1421
30-44		197	124	63%	73	37%	
45-59		185	126	68%	59	32%	
Over 60		135	95	70%	40	30%	
Continuous Age					(T-Test P-Value)		0.0025
**Length of time in current district**							
> 10 years		497	325	65%	172	35%	0.3508
6-10 years		79	51	65%	28	35%	
≤ 5 years		74	42	57%	32	43%	
**Birth Region**							
Rainforest		247	165	67%	82	33%	0.5949
Andean		279	176	63%	103	37%	
Coastal		41	25	61%	16	39%	
**Individual Economic Activity Income (Peruvian New Soles)**							
No income		253	157	62%	96	38%	0.0777
200-3000		92	67	73%	25	27%	
3100-7200		95	53	56%	42	44%	
7300-12000		124	87	70%	37	30%	
12100- 67500		86	54	63%	32	37%	
**Economic Activity**							
Agricultural Work	No	473	294	62%	179	38%	0.0613
	Yes	177	124	70%	53	30%	
Timber Industry	No	618	401	65%	217	35%	0.1757
	Yes	32	17	53%	15	47%	
Brazil Nut Harvesting	No	627	400	64%	227	36%	0.1550
	Yes	23	18	78%	5	22%	
Fisherman	No	640	408	64%	232	36%	0.0176
	Yes	10	10	100%	0	0%	
Merchant	No	606	394	65%	212	35%	0.1616
	Yes	44	24	55%	20	45%	
Miner	No	638	409	64%	229	36%	0.4352
	Yes	12	9	75%	3	25%	
Professional/Technical	No	633	408	64%	225	36%	0.6325
	Yes	17	10	59%	7	41%	
Employee of a store/restaurant/warehouse	No	626	399	64%	227	36%	0.1216
	Yes	24	19	79%	5	21%	
Taxi driver/transportation	No	620	404	65%	216	35%	0.0389
	Yes	30	14	47%	16	53%	
Other work	No	588	377	64%	211	36%	0.7530
	Yes	62	41	66%	21	34%	
**District**							

Huepetuhe	38	15	39%	23	61%	
Inambari	106	54	51%	52	49%	
Laberinto	52	33	63%	19	37%	
Tambopata	146	101	69%	45	31%	0.0006
Las Piedras	170	118	69%	52	31%	
Tahuamanu	57	37	65%	20	35%	
Iberia	33	23	70%	10	30%	
Inapari	48	37	77%	11	23%	
Rural Community	431	288	67%	143	33%	0.0606
Urban Community	219	130	59%	89	41%	

Table S3. Individual Birth Region by Selected Characteristics.

Characteristic	Individual Birth Region								Chi-Sq
	Rainforest		Andean		Coastal		Total		P-Value
	n	%	n	%	n	%	n	%	
Household fish consumption									
Never	0	0%	12	92%	1	8%	13	4%	
Monthly	57	35%	95	58%	13	8%	165	45%	<.0001
Weekly	78	53%	58	40%	10	7%	146	40%	
Daily	28	70%	11	28%	1	3%	40	11%	
Fisherman in household	10	77%	2	15%	1	8%	13	4%	0.047
No fisherman in household	154	44%	174	49%	24	7%	352	96%	
Uses well water	43	42%	45	44%	14	14%	102	28%	0.005
Uses other water source	121	46%	131	50%	11	4%	263	72%	
Mining occupation	5	63%	2	25%	1	13%	8	2%	0.069
No mining occupation	159	45%	174	49%	24	7%	357	98%	
In and adjacent mining	31	34%	58	63%	3	3%	92	25%	0.003
No in and adjacent mining	133	49%	118	43%	22	8%	273	75%	

Table S4. Trace Element Concentrations and Differences in Trace Element by Nail Type for Adults in the IMAS Study Population, Madre de Dios, Peru (n = 399).

Trace Element	Fingernail		Toenail		Total		Outlier Status (Chi-Sq)	Logged Element (ANOVA)
Total	283	71%	116	29%	399	100%	P-Value	P-Value

Arsenic	Non-Outlier	270	71%	111	29%	381	95%	0.9015	<.0001
	Outlier	13	72%	5	28%	18	5%		
Cadmium	Non-Outlier	265	72%	105	28%	370	93%	0.2753	0.2844
	All	18	62%	11	38%	29	7%		
Mercury	Non-Outlier	258	69%	115	31%	373	93%	0.0034	0.0113
	Outlier	25	96%	1	4%	26	7%		
Lead	Non-Outlier	242	70%	106	30%	348	87%	0.1114	0.0998
	Outlier	41	80%	10	20%	51	13%		

Table S5. Trace Element in Nails Retest Percent Differences, Adults in the IMAS Study Population, Madre de Dios, Peru.

Element	Mean	Min	Max
As	16.5	0.8	45.3
Cd	25.7	2.9	88.7
Hg	13.9	0.7	34.9
Pb	19.4	1	52.7

*Absolute Values of Percent Differences

Supplementary Figures

Sample Design: Households in MDD located within 10 km of the IOH having lived at their residence for 6 months prior to the survey

Stage 1: 10 urban and 38 rural localities selected

Stage 2: 636 Households (HH) selected via PPS:

- 18 HH per urban locality
- 12 per rural locality

2011 Sample: 558 HH Approached

- 2 rural localities no longer existed- 24 HH
- 31 HH declined (2/3 were in (illegal) mining areas)
- 8 HH abandoned
- 32 No head of HH present each of 3 times approached

487 HH Surveyed (RR All Surveys: 87%)

- 433 HH completed surveys (RR Completed Surveys 78%)
- 54 HH Incomplete surveys
- 1664 individuals

2014: 487 Households Approached

310 Households Surveyed (64% RR all surveys): 231 HH Completed surveys (47% RR completed surveys) + 79 HH Incomplete surveys

1208 people in the HHs:

- 1021 Present
    - 650 Adults – 309 Female / 341 male
    - 371 children (184 female/ 187 male)
- 178 Individuals not present
- 9 deceased

418 Adults gave nail samples (200 males/ 218 females) from 273 households (64% of 650 adult participants).

Figure S1. Study Flow Chart
